# Probiotics in the Management of Atopic Dermatitis for Children: A Case-Based Review

**DOI:** 10.1155/2020/4587459

**Published:** 2020-12-07

**Authors:** Ashila Putri Disamantiaji, Endang Farihatul Izza, Muhamad Faza Soelaeman, Tannia Sembiring, Melva Louisa

**Affiliations:** ^1^Faculty of Medicine Universitas Indonesia, Jakarta, Indonesia; ^2^Department of Pharmacology and Therapeutics, Faculty of Medicine Universitas Indonesia, Jakarta, Indonesia

## Abstract

**Background:**

Atopic dermatitis or eczema is one of the most common dermatologic problems, especially in children. Several studies have hypothesized that alteration of gut-colonizing microbes might have induced and conditioned the development of the disease. Thus, modulation of microbial diversity and abundance might help alleviate symptoms and conditions for patients. Given the ability of commensal and symbiotic microorganisms in modulating the immune system, probiotics administration has been studied in previous research in the management of eczema. However, until today, there are conflicting results between studies making inconclusive recommendations towards probiotics supplementation in the management of atopic dermatitis. This case-based review was done to assess and evaluate the therapeutic efficacy of probiotics supplementation in the management of eczema in children.

**Method:**

An electronic database search was conducted in PubMed-NCBI, Cochrane, EBSCO, ProQuest, and SCOPUS in March 2020. Individual studies and reviews were then gathered for screening using predetermined inclusion and exclusion criteria. The included studies were then critically appraised for their validity and importance.

**Result:**

A total of 5 studies, all of which were RCTs, were included in this review. Out of all the studies included, 4 showed no clinically significant improvements in using probiotics in the management of eczema in children as they did not pass the minimal clinically important difference (MCID) of eczema severity as determined by SCORAD (SCORing Atopic Dermatitis).

**Conclusion:**

Supplementation of probiotics in the management of eczema in children does not show a clinically relevant difference vs. standard treatment in reducing eczema severity.

## 1. Introduction

A 27-year-old mother with her 7-year-old daughter came to a general practitioner (GP) with a chief complaint of itching on her daughter's face. During the physical examination, she appeared to have rashes on her cheeks, forehead, and scalp, which had been apparent starting two weeks ago. The rashes then spread to her knees, elbows, and trunks, causing the daughter to lose sleep and hindering her day-to-day activities. The GP diagnosed her with atopic dermatitis and planned to prescribe her a topical corticosteroid. The mother then mentioned that she was also diagnosed with atopic dermatitis since she was little. Several severe exacerbations had occurred and caused her to consume oral corticosteroids. After she did some research on the Internet, she heard that using corticosteroids for a long time would bring detrimental health effects. She was worried that her daughter might have to consume oral corticosteroids for a long time. She then asked the GP whether there would be any additional or alternative treatments with minimum side effects that would help alleviate her daughter's symptoms without solely relying on corticosteroids. The GP has previously read articles reporting altered microbiota composition in patients with allergies, autoimmune diseases, or other immune-mediated diseases. Therefore, the GP would like to find out whether recommending probiotics would result in a better outcome in terms of the patient's symptoms.

Atopic dermatitis (AD), also known as atopic eczema, is a long-lasting, recurrent, pruritic, and inflammatory eczematous eruption that is common in children [1]. It is a significant burden on global public health, affecting up to 20% of children and about 3% of adults worldwide. The incidence of AD has increased by 2- to 3-fold during the past decades in industrialized countries [2]. The cause of AD is complex and not fully understood but is likely to be multiple factors, including genetic, environmental, and socioeconomic [1]. AD poses a substantial burden on the patients' quality of life because of sleep deprivation due to itchiness, time of care, and financial cost [3, 4].

Individualized therapy to treat AD should be applied with regard to the patients' age, severity, and distribution of the lesion. Fundamentally, therapy of AD can be categorized into three options: basic, standard medical, and adjuvant treatment. The basic procedure is cutaneous hydration, elimination of triggering factors such as allergens and irritants, and maintaining good personal hygiene. It was given to reduce dryness with emollient, which often relieves pruritus. Standard medical treatments are given according to the severity of the disease [4, 5]. The severity of AD sign and symptoms can be measured objectively by the clinician and subjectively by patient- or family-reported questionnaires, such as scoring atopic dermatitis (SCORAD), eczema area and severity index (EASI), and visual analog scale for pruritus (VASP), which are comparable to each other [4].

In patients with mild symptoms, topical medications are the first line of treatment. If essential and topical treatments fail, a systemic medication may be necessary. However, the International Eczema Council had stated that systemic corticosteroid is not recommended for children. Hence, adjuvant therapy is sometimes considered if the symptoms are uncontrolled by adequate basic treatment [4, 5].

There are plethora options of adjuvant therapy for AD, including probiotics. The immune system and inflammation had substantial contributions in the clinical manifestations of AD. The disruption in the epidermal barrier permits increased penetration of external antigens and initiates skin inflammation. This promotes the interaction of foreign antigens with the antigen-presenting cells and immune effector cells that may prompt the elicitation of systemic immune responses via a positive feedback mechanism between Th2/Th17 inflammation and barrier dysfunction [6].

Recent studies have shown that gut microbiomes have been implied in the development and shaping of the host immune system. Gut dysbiosis-alterations of microbial diversity and abundance due to lifestyle and diet correspond to the impaired inflammatory response to a particular antigen. This phenomenon has been demonstrated in patients with AD [7]. In general, it is known for its short-chain fatty acid- (SCFA-) producing capability. *Bifidobacterium*, *Propionibacterium*, *Coprococcus*, *Blautia*, and *Eubacterium* are significantly reduced in patients with AD [8–10]. Since SCFA can induce regulatory T (Treg) cell expansion, reduction of SCFA may result in a shift of Treg-Th17 balance towards Th17, T helper cell known for its role in orchestrating proinflammatory immune response. Repopulating gut microbiota has been suggested to be an alternative mechanism to treat various immunologic diseases, including AD [11]. However, the approach is still controversial [12]. The purpose of this article is to determine whether giving probiotics to children with AD would result in improved symptoms based on currently available evidence.

## 2. Methodology

### 2.1. Literature Search

A comprehensive search was done on five databases: PubMed, Cochrane, EBSCO, ProQuest, and SCOPUS in March 2020. The keywords used were as follows: “probiotics,” “dermatitis,” “eczema,” and “treatment” from which synonyms were used based on MeSH terms to expand its reach. Boolean operators were then incorporated to form a complete search. The results were then screened for duplicates using the systematic review accelerator-deduplication module (SRA-DM). This citation-screening program has been validated and demonstrated to consistently have higher sensitivity and specificity compared to other reference management software's deduplication features [13]. The auto-deduplication process done through SRA-DM was followed by manual hand-searching to remove the remaining undetected duplicates. The final inclusion of studies was done by inspecting each full manuscript and evaluating whether or not the study meets the selection criteria.

### 2.2. Eligibility Criteria

We included randomized controlled trials and systematic reviews of meta-analysis with the target population of children up to 18 years old diagnosed with atopic dermatitis who were (or not) undergoing AD treatment. We only included studies that use SCORAD, EASI, or VASP as the outcome measure. We did an additional hand search of studies mentioned in the systematic review which explain probiotics use in children with eczema compared to no treatment, placebo, or another intervention with no probiotics. Articles were excluded if the studies used heat-killed bacteria instead of viable microorganisms as their interventions and if the study used any language other than English.

### 2.3. Critical Appraisal

The Centre for Evidence-Based Medicine (CEBM) appraisal tools were utilized to assess the quality of the included studies. For RCTs, the appraisal was done by assessing the papers' quality from the domains of validity (randomization, blinding, and comparability) and clinical importance. To determine the importance of each studies' result, we compared the mean difference of each SCORAD score at the end of the intervention period to a predefined minimal clinically important difference (MCID), which is 8.7 [14].

## 3. Results

### 3.1. Search Findings

**Figure 1:**
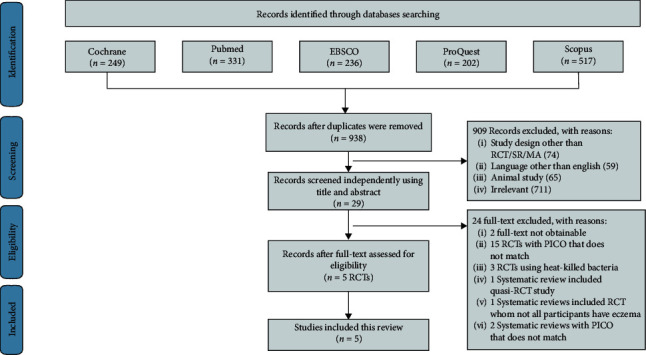
PRISMA flowchart describing the steps of study selection.

Using search strategies, as mentioned in Table 1, 1535 relevant studies were identified through five electronic database exploration, from which duplicates were removed, resulting in 938 remaining studies. The entire process of searching databases, deduplicating results, and study selection is shown in Figure 1. As shown in Figure 1, a total of 909 studies were removed due to the reason of study selection criteria (*n* = 74), language used was not English (*n* = 59), animal studies (*n* = 65), and other reasons (*n* = 711), leaving 29 studies to be screened. Twenty-four studies were then excluded, from which two were not available, and 22 others were not compatible with our PICO. As a result, the 5 RCTs retrieved from database searches were utilized in this article.

### 3.2. Appraisal Results

Based on the CEBM RCT appraisal tool, only two out of five RCTs fulfilled the validity criteria entirely. The three remaining studies had varied results on the validity assessment. The summary of the validity assessment is shown in Table 2. Randomization was thought to be done adequately by three studies (Han et al. [19], Navarro-Lopez et al. [15], and Yang et al. [17]). The three studies allocated participants using computer-generated lists of random numbers. While Woo et al. [18] and Prakoeswa et al. [16] mentioned that they randomized their participants into two arms of intervention, they did not further describe and clarify the method used in generating random sequences.

All five RCTs achieved a similarity in baseline characteristics for both the groups. No statistically significant difference was observed between both the placebo and probiotic groups in terms of baseline sociodemographic and clinical characteristics in all trials. Variables that are deemed to be clinically relevant, such as age, initial SCORAD score, and duration of illness, were also similar between the two groups for all trials. In the trial done by Woo et al. [18], boys were underrepresented in the placebo group. In contrast, in the trial done by Prakoeswa et al. [16], the placebo group was dominated by male participants, and female participants were dominated the probiotic group. Since gender does not imply atopic dermatitis' pathogenesis—and thus we judged gender to be of no clinical importance—the baseline sociodemographic characteristics of probiotic and placebo groups were considered to be similar in both the trials.

Among five RCTs, three trials (Han et al. [19], Navarro-Lopez et al. [15], and Prakoeswa et al. [16]) did intention-to-treat analysis. Prakoeswa et al. did not report any participants with noncompliance or lost-to-follow-up, and all participants were retained within their respective groups after the randomization process had been undertaken. Measured outcomes up to the point of the last follow-up for three lost-to-follow-up participants (week 4) were still reported, and those three participants with the missing outcome at week 12 were not included in the denominator of effect estimate's calculation. [16] Han et al. did both the per-protocol and intention-to-treat analyses [19]. Randomized participants that did not adhere to the protocol were not included in the analyses of two trials (Woo et al. [18] and Yang et al. [17]).

Four RCTs did blinding (Han et al. [19], Navarro-Lopez et al. [15], Woo et al. [18], and Yang et al. [17]) by making the probiotic and placebo preparations identical. Prakoeswa et al. [16] did not elaborate on blinding. Participants in all RCTs were equally treated in both the groups. Four out of the five included studies did not find a clinically significant difference in the use of probiotics to improve AD symptoms by the end of the trial (6 weeks in the study of Yang et al. and 12 weeks in the remaining studies), which was shown by the mean difference of the studies achieving the minimal clinically important difference (MCID). In the clinically significant research done by Navarro-Lopez et al., statistical significance was also obtained between the probiotic and placebo groups [15–19].

### 3.3. Types of Probiotics Used

In three RCTs (Prakoeswa et al. [16], Navarro-Lopez et al. [15], and Woo et al. [18]), probiotics were administered alongside the participants' standard treatment of eczema based on guidelines. The probiotics in those studies were administered orally, from which the study conducted by Prakoeswa et al. [16] gave a *Lactobacillus plantarum* IS-10506, Navarro-Lopez et al. [15] gave a probiotics combination of *Bifidobacterium lactis* CECT 8145, *Bifidobacterium longum* CECT 7347, and *Lactobacillus casei* CECT 9104, while Woo et al. [18] used *L. sakei* KCTC 10755BP. Yang et al. [17] used a mixture of probiotic strains, including *L. casei, L. rhamnosus*, *L. plantarum*, and *Bifidobacterium lactis* suspended in glucose anhydrous crystalline powder. Unlike the three RCTs mentioned above, all participants from the trial were told to stop using topical corticosteroids, oral antihistamines, topical calcineurin inhibitors, or any probiotic-containing product starting from two weeks before the commencement of the study. Han et al. [19] gave an *L. plantarum* CJLP133, which was administered orally without the use of topical corticosteroids during the study period. In three RCTs done, respectively, by Woo et al. [18], Yang et al. [17], and Han et al. [19], participants' parents received instructions on the use of topical emollients and were trained on the proper bathing and skincare practice.

### 3.4. Probiotics and Improvement of Eczema Symptoms

Four out of five included RCTs demonstrated that the primary outcome of all of the studies was similar and monitored eczema symptoms through SCORAD index assessment at different time points. Prakoeswa et al. [16] obtained the SCORAD change at the 2nd week and 8th week for the second measurement and followed up at the 12th week after the intervention. In contrast, Navarro-Lopez et al. [15] described the change in the 4th week, 8th week, and 12th week after the intervention. Also, Woo et al. [18] did measure the SCORAD at the 6th week and followed up at the 12th week after treatment. Han et al. [19] measured the SCORAD change during the 16-week study, which included a 12-week trial, a 2-week washout period, and 2-week after discontinuation of either probiotics or placebo administration. Unlike the four studies above, Yang et al. [17] used EASI and VASP to assess improvement in eczema symptoms at 0- and 6-week time points—the time limit for the intervention period. The treatment follow-ups were done at different frequencies and time points. Thus, the 95% CIs described are based on the SCORAD or EASI and VASP scores at the endpoint of each study, which was at the 6th week after treatment for the study by Yang et al. and the 12th week after treatment for the four remaining studies [15–19].

Only one study showed a clinically and statistically significant impact of delivering probiotics to the symptoms of eczema in children. Navarro-Lopez et al. found a significant improvement of symptoms in the participants who received probiotics as represented by 17.8 points lower score, passing the MCID score of 8.7 [15]. On the contrary, the remaining studies did not achieve clinical significance even though statistical significance was achieved. Prakoeswa et al. [16] showed that the mean difference between the two groups did not obtain MCID although a statistically significant decrease in the SCORAD score (placebo vs. probiotic) was observed (*p*=0.000). Woo et al. [18] found a substantial reduction in the pretreatment-adjusted SCORAD total scores at week 12. Overall, SCORAD measured in the probiotic group was 28.8 (95% CI, 25.1–32.4) and 35.8 (31.9–39.8) in the placebo group. However, the mean difference between the two groups by week 12 only resulted in a SCORAD of 7, which did not pass the MCID. Han et al., which used both intention-to-treat (ITT) and per-protocol analysis, show a statistically significant decrease in the mean difference of probiotic groups for both scenarios. In ITT analysis, the total SCORAD score for the probiotics group was 20.4 ± 11.8 (16.9 to 23.9) and 25.6 ± 11.6 (22.0 to 29.2) for the placebo group (*p*=0.044). However, the mean reduction between the two groups at the endpoint of the trial did not pass MCID. The summary of importance is shown in Table 3.

On the contrary, one study by Yang et al. [17] demonstrated no statistically significant difference in EASI and VASP between probiotic and control groups at the end of the treatment. When compared with the initial EASI and VASP scores before the intervention commencement (baseline vs. endpoint of trial), significant improvement (*p* < 0.01) in EASI and VASP scores was observed in the group treated with probiotics; however, this positive outcome (*p* < 0.01) was also found in the placebo group. The mean difference between EASI score for both the groups after six weeks did not reach its MCID, which was already predetermined in a previous study to be a 6.6-point change in the mean score [14]. To date, no MCID for the use of VASP in atopic dermatitis patients has been reported. Nevertheless, we judged that a 0.5-point difference in VASP scores between the both groups to be of no clinical importance. The summary of all articles is shown in Table 4.

## 4. Discussion

Up to date, the place of probiotics for the treatment of eczema in children is still controversial. Thus, this evidence-based case report was done to determine whether the use of probiotics is recommended in alleviating the severity of dermatitis in children.

Across all included studies, only Navarro-Lopez et al. had significant clinical importance as indicated by the mean difference between two groups, which passed the MCID score of 8.7. We assume that the main reason why Navarro-Lopez et al. showed a significant effect was the use of a combination of several probiotic strains in their study design. However, previous studies found that it gives better beneficial effects when compared to a single probiotic regimen, especially when both lactic acid bacteria, *Lactobacillus* sp. and *Bifidobacteria* sp., were combined [20]. The most evident differences between Navarro-Lopez et al.'s from the other four studies were the duration of treatments, the age of the children, and the severity of AD. Although the other four studies had shown a tendency of a better efficacy outcome in probiotic groups, none had achieved MCID.

Following this argument, the study by Yang et al. should also show a substantial effect in this study. However, as mentioned in the referred study, they also consider the lack of diet limitation in their study protocol as a possible explanation behind their results. Their population is thought to have a high amount of fermented food in their diet, which might also contain live microorganisms [17]. On the contrary, the study by Navarro-Lopez et al. restricts patients' diets by only including those currently consuming a high-quality Mediterranean diet, with a Mediterranean Diet Quality Index (KIDMED) score of more than 7 [15].

Aside from the absence of restriction on consuming fermented food/beverages in Yang et al.'s study, a considerable reduction in total SCORAD score at week six from baseline observed in both probiotics and placebo group might result from the effect of proper bathing and emollient use and not from the standard of care regiment. It can be predicted as all participants were told to stop using topical corticosteroids, topical calcineurin inhibitors, and oral antihistamines starting from two weeks before the administration of probiotics [15]. Meanwhile, four other studies by Han, Navarro-Lopez, Prakoeswa, and Woo still allowed the use of topical corticosteroids [15, 16, 18, 19]. The placebo groups in trials done by Navarro-Lopez et al. and Prakoeswa et al. also received standard therapy [15, 16]. Concomitant use of conventional therapy/topical corticosteroid, proper bathing practice, and emollient use that were implemented in both probiotic and placebo groups might serve as an explanation on how the mean difference of total SCORAD score or EASI score in four trials (those by Han, Navarro-Lopez, Prakoeswa, and Woo) did not achieve the MCID [15, 16, 18, 19].

Several limitations should be considered from the studies. Several things that may make a comparison between studies difficult are differences in bacterial strains used, formulation, doses given to the participants, and the patients' clinical severity before the initiation of the study. Although both of the strains' class, *Bifidobacterium* and *Lactobacillus,* are typically found in fermented foods and supplements, specific formulation and conditions required for those strains as used in the studies are challenging to mimic in the real life, resulting in possibilities of different results in patients. The doses are taken by participants in the studies, both in size and frequency, are also varied. Such amount and rate of consumption might not be applicable in real life, thus resulting in a possible subthreshold effect for patients and giving not as desired results.

The small number of participants enrolled in the studies should also be considered as a limitation as they could lower the statistical power of the study. The largest number of participants was only 108 (with only 83 accounted for analysis) in [19], and the smallest was 22 in [16]. High drop-out rate in several studies such as those by Han et al. could diminish the statistical power of the study. Besides, differences in participants' severity could result in a generalized outcome that might not be applicable for patients in actual clinical settings. The different outcomes measured should also be taken into consideration. Although most of the studies measured the SCORAD index as their primary outcome, Han et al. and Yang et al. used EASI, which leads to different interpretations [15–19].

The patient in the clinical scenario has no significant difference regarding race and age compared to four out of the five studies which are done in Asia. However, compared to the study by Navarro-Lopez, whose participants were European, gut microbiota diversity might be different. Probiotics are commercially available and affordable in Asian countries [21]. Regarding the harm and benefit of probiotics, no adverse effects were reported in the studies included. However, Boyle et al. reported 42 cases of proven and suspected probiotics-related sepsis but then suggested that Bifidobacteria have a better safety profile compared to other probiotic strains [22].

## 5. Conclusion

Probiotics show no significant effect on improving clinical symptoms in the management of atopic dermatitis. Up to date, the addition of probiotics to the current atopic dermatitis treatment is not recommended. Efforts to homogenize regimens of bacterial strains used and doses given in future studies are needed to establish relevant results of probiotics use for eczema patients in clinical settings.

## Figures and Tables

**Table 1:** Search strategy undertaken.

Database	Search strategy	Hits
Cochrane	Eczema OR dermatitis in title abstract keyword AND therap^*∗*^ OR treatment in title abstract keyword AND probiotic in title abstract	249
PubMed	(((((Eczema (MeSH terms)) OR eczema (title/abstract)) OR dermatitis (title/abstract))) AND ((probiotic (MeSH terms)) OR probiotic (title/abstract))) AND (((treatment (title/abstract)) OR therap^*∗*^(title/abstract)) OR therapeutics (MeSH terms)	331
EBSCO	(Eczema^*∗*^ OR dermatitis) AND (probiotic^*∗*^) AND (therap^*∗*^ OR management OR treat^*∗*^) NOT prevention NOT risk	236
ProQuest	(ti(eczema^*∗*^ OR dermatitis OR atop^*∗*^) OR ab(eczema^*∗*^ OR dermatitis OR atop^*∗*^)) AND (ti(probiotic^*∗*^) OR ab(probiotic^*∗*^)) AND (ti(treatment^*∗*^ OR therap^*∗*^) OR ab(treatment^*∗*^ OR therap^*∗*^)) NOT (ti(prevention) OR ab(prevention))	202
	NOT (asthma AND animals AND food hypersensitivity AND mice AND food allergies AND obesity AND pregnancy AND diarrhoea AND animal models AND feces AND gastrointestinal diseases AND biomarkers AND immunotherapy AND vitamin d AND adult AND anaphylaxis AND cow's milk)	
	NOT (news AND report AND general information AND commentary AND case study AND correspondence AND instructional material/guideline AND company profile AND editorial AND literature review)	
Scopus	TITLE-ABS-KEY (eczema^*∗*^ OR atop^*∗*^ OR dermatitis) AND (probiotic^*∗*^) AND (treatment^*∗*^ OR therap^*∗*^) AND NOT (prevention) AND NOT (asthma OR ibs)	517

**Table 2:** Validity appraisal of RCTs based on CEBM critical appraisal tools.

Author	No. of participants	Level of evidence	Validity^a^				
			Randomization	Baseline similarity	Equally treated	Intention to treat	Blinding
Navarro-Lopez et al. [15]	50	2	+	+	+	+	+
Prakoeswa et al. [16]	22	2	?	+	+	+	?
Yang et al. [17]	70^b^	2	+	+	+	−	+
Woo et al. [18]	88	2	?	+	+	−	+
Han et al. [19]	108	2	+	+	+	+	+

^a^Validity demonstrated by: +, clearly stated; −, not done; ?, not clearly stated. ^b^Out of 100 initial participants, only 70 participants finished the study. The conclusion was made based on the per-protocol analysis.

**Table 3:** Importance appraisal of RCTs based on CEBM critical appraisal tools.

Importance						
Author	Parameters for efficacy endpoints	MCID	Mean total SCORAD score in the probiotic group at the trials' endpoint ± SD (CI 95%)	Mean total SCORAD score in the placebo group at the trials' endpoint ± SD (CI 95%)	Mean difference between the two groups (probiotic-placebo) (CI 95%)	Comments
Navarro-Lopez et al. [15]	SCORAD^a^	8.7	6.8	24.4	17.8^b^	The mean difference between the two groups achieved MCID
Prakoeswa et al. [16]	SCORAD^a^	8.7	18.53 ± 14.200	22.04 ± 8.817	3.507 ± 4.96 (95% CI 1.44 to 5.58)	The mean difference between the two groups did not achieve MCID
Yang et al. [17]	EASI^a^ and VASP	6.6 for EASI and N/A for VASP	4.7 ± 3.4 (EASI) and 3 (VASP)^c^	4.5 ± 4.7 (EASI) and 2.5 (VASP)^c^	−0.2 ± 0.86 (95% CI, −1.74 to 2.14 for EASI)^d^	Mean difference between the two groups did not achieve MCID
Woo et al. [18]	SCORAD^a^	8.7	28.8 ± 11.92 (25.1–32.4)	35.8 ± 11.75 (31.9–39.8)	−7 ± 22.98 (95% CI, −12.2 to −1.8)	Mean difference between the two groups did not achieve MCID
Han et al. [19]	SCORAD^a^	8.7	20.4 ± 11.8 (16.9 to 23.9)	25.6 ± 11.6 (22.0 to 29.2)	−5.2 ± 27.93 (95% CI, −10.24 to −0.16)	Mean difference between the two groups did not achieve MCID

^a^MCID for these studies was obtained from a study that measured the MCIDs of SCORAD, EASI, and POEM for atopic eczema by Schram et al. [14]. ^b^Data were not adequate to calculate the confidence interval. ^c^Result was reported using a nonparametric statistic, and no confidence interval was reported. Converted result into parametric data was retrieved from a systematic review [15]. ^d^Mean reduction between two groups was extracted from a systematic review that had transformed the result into parametric data beforehand. MCID for VASP that is specific for eczema has never been investigated [1].

**Table 4:** Summary of included studies.

Author	Study population	Probiotics (types, dose)	Outcome measure	Result
Navarro-Lopez et al. [15]	Children, 4–17-year-old with moderate atopic dermatitis	*Bifidobacterium* lactis CECT 8145, *B. longum* CECT 7347, and *Lactobacillus casei* CECT 9104 freeze-dried powder in a capsule, twelve weeks, once daily	SCORAD index	The intervention group who received probiotic has significant improvement both statistically (*p* < 0.05) and MCID (SCORAD > 8.7)
Prakoeswa et al. [16]	Children, 0–14-year-old with atopic dermatitis who met the Hanifin–Rajka diagnostic criteria, age-related total serum IgE levels of 10–15 years > 200 IU/mL, 6–9 years > 90 IU/mL, 1–5 years > 60 IU/mL, <1 year > 1.5 IU/mL	*L. plantarum* IS-10506 at the dose of 10^10^ cfu/day for 12 weeks in microencapsulated form	SCORAD index	A statistically significant decrease of SCORAD score in the probiotic group was observed (*p* ≤ 0.001); however, MCID was not achieved (SCORAD < 8.7)
Yang et al. [17]	Children, 2–9-year-old with mild to moderate atopic dermatitis (SCORAD score ≤40) who met the Hanifin–Rajka diagnostic criteria without any chronic underlying disease, acute GI infection, or past exposure to commercial probiotics/systemic corticosteroid/antibiotic/immunosuppressive agent/Chinese herbal therapies 4 weeks prior to enrollment	Mixture of *L. casei*, *L. rhamnosus*, *L. plantarum,* and *B*. *lactis* in glucose anhydrous crystalline powder derived from corn starch; a single dose preparation containing 1 × 10^9^ cfu of each bacterial strain, given for 6 weeks, twice daily	EASI (eczema area and severity index) and VASP (visual analog scale for pruritus) scores	Statistically significant decrease in EASI and VASP scores before and after treatment in both probiotic and control groups
				No statistically significant difference between probiotic and control groups in EASI and VASP scores at week 6
Woo et al. [18]	Children, 2–10-year-old with moderate and severe AEDS (atopic eczema dermatitis syndrome)	Microcrystalline cellulose (1.76 g) as a carrier of freeze-dried *L. sakei* KCTC 10755BP; a dose of 5 × 10^9^ cfu of *L. sakei* was given twice daily	SCORAD index	SCORAD total score-adjusted pretreatment values were lower after probiotic vs placebo treatment at week 12 (*p*=0.01); there was a greater improvement in mean disease activity with probiotic (31%) vs. placebo use (13%), *p*=0.008; however, MCID was not achieved
Han et al. [19]	Children, 1–13-year-old presenting with AD with SCORAD ranged from 20–50	*L. plantarum* CJLP133 at the dose of 0.5 × 10^10^ given twice daily for 12 weeks	SCORAD index	Mean change of the SCORAD score from baseline in probiotic group was greater than in placebo group, both in ITT (*p*=0.002) and PP analysis (*p*=0.002); however, MCID was not passed

## Data Availability

The data supporting this manuscript can be obtained from the corresponding author upon request.
